# Associations between metabolomic scores and clinical outcomes in hospitalized COVID-19 patients

**DOI:** 10.1007/s11357-025-01591-z

**Published:** 2025-03-11

**Authors:** Jens A. Venema, Anna Kuranova, Daniele Bizzarri, Simon P. Mooijaart, Angele P. M. Kerckhoffs, Kitty Slieker, Evertine J. Abbink, Harmke A. Polinder-Bos, Eline Slagboom, Geeske Peeters, the COOP consortium, Jacobijn Gussekloo, Karel G. M. Moons, Maarten van Smeden, René J. F. Melis, Petra J. M. Elders, Jan Festen

**Affiliations:** 1 Department of Geriatric Medicine, Radboud University Medical Center, Nijmegen, the Netherlands; 2 Department of Biomedical Data Sciences, Section of Molecular Epidemiology, Leiden University Medical Center, Leiden, the Netherlands; 3 Department of Internal Medicine, Section of Gerontology and Geriatrics, Leiden University Medical Center, Leiden, the Netherlands; 4 LUMC Center for Medicine for Older People, Leiden University Medical Center, Leiden, the Netherlands; 5 Department of Internal Medicine, Department of Geriatrics, Jeroen Bosch Hospital, Den Bosch, the Netherlands; 6 Department of Internal Medicine, Bernhoven Hospital, Uden, the Netherlands; 7 Department of Internal Medicine, Radboud University Medical Center, Nijmegen, the Netherlands; 8 Section of Geriatrics, Department of Internal Medicine, Erasmus MC University Medical Centre, Rotterdam, the Netherlands; 9 Radboudumc Alzheimer Centre, Radboud University Medical Center, Geert Grooteplein Zuid 10, Route 925, Postbus 9101, 6500 HB Nijmegen, The Netherlands

**Keywords:** Metabolomics, COVID-19, Frailty, Ageing

## Abstract

The disease course and outcome of COVID-19 greatly varies between individuals. To explore which biological systems may contribute to this variation, we examined how individual metabolites and three metabolic scores relate to COVID-19 outcomes in hospitalized COVID-19 patients. The metabolome of 346 patients was measured using the 1H-NMR Nightingale platform. The association of individual metabolomic features and multi-biomarker scores, i.e. MetaboHealth, MetaboAge, and Infectious Disease Score (IDS) (higher scores reflect poorer health), with in-hospital disease course, long-term recovery, and overall survival were analyzed. Higher values for the metabolites phenylalanine (HR = 1.33, CI = 1.14–1.56), glucose (HR = 1.37, CI = 1.16–1.62) and lactate (HR = 1.38, CI = 1.16–1.63) were associated with mortality. For all three metabolic scores, higher scores were significantly associated with higher odds of a poorer in-hospital disease course (MetaboHealth: OR = 1.61, CI = 1.29–2.02; ΔMetaboAge: OR = 1.42, CI = 1.16–1.74; IDS: OR = 1.55, 1.25–1.93) and with overall survival (MetaboHealth: HR = 1.57, CI = 1.28–1.92; ΔMetaboAge: HR = 1.34, CI = 1.15–1.57; IDS: HR = 1.56, CI = 1.27–1.93). MetaboHealth and ΔMetaboAge showed a stronger association in younger patients (< 70 yrs.) than older patients. No clear patterns were found in associations between the three scores and measures of long-term recovery. In conclusion, the heterogeneity in disease course after SARS-COV2 infection may be explained either by generic biological frailty reflected by the three metabolomics scores or by glycemic control (glucose, lactate) and respiratory distress (phenylalanine).

**Supplementary Information:**

The online version contains supplementary material available at 10.1007/s11357-025-01591-z.

## Introduction

The COVID-19 pandemic has had an unprecedented impact on the well-being of all people in society. Over 7 million people have died from COVID-19 worldwide, and 65 million patients are estimated to suffer from long COVID [1, 2]. Great heterogeneity is observed in the disease course, both during the active disease phase and the recovery phase. A better understanding of the mechanisms underlying this heterogeneity is needed to inform prognosis and personalized medicine. Several risk factors emerge as pivotal factors for severe disease course after COVID-19 infection, including older age [3, 4]. In the past decade, molecular biomarkers, such as metabolomic assays, have been explored for a range of age-related diseases and frailty and may provide indications for which biological mechanisms drive disease heterogeneity.

Among various established metabolomic profiles, several composite scores generated in epidemiological biomarker research appear particularly promising for their role and predictive ability in the context of COVID-19. These scores provide a comprehensive view of an individual’s health status, transcending the limitations of traditional single biomarkers. In this study, in addition to 62 individual metabolic features, we focus on three key metabolomic scores: the MetaboHealth score, the MetaboAge score, and the Infectious Disease Score [5–7]. The algorithms for these scores were developed in large epidemiological datasets and were selected for their ability to predict aspects of health and aging that are crucial for understanding the complexities of COVID-19, specifically in older adults.

MetaboHealth was designed to indicate global vulnerability in older individuals by optimizing it for the risk prediction of all-cause mortality with better accuracy than generally measured clinical risk variables [5]. The score demonstrated to be an interesting quantification of a person’s general health and cognitive decline, and a stronger predictor of frailty than MetaboAge [5, 8, 9]. The MetaboAge score was designed to indicate a measure of biological age since it calculates the difference between metabolic age and calendar age which indicates especially the risk of future cardiovascular disease, mortality, and decreased functionality in the oldest old [7]. The Infectious Disease Score was designed to indicate susceptibility to infectious disease by optimizing it for the risk prediction of pneumonia in the UK Biobank and indicates the risk of hospitalization due to pneumonia [6]. It also predicts the risk of COVID-19 independent of already existing disease [6]. All scores were generated in large datasets and validated in independent studies. Previous studies have shown the prognostic value of these three metabolic scores in different contexts, such as testing the association between day and night shift workers and predicting recovery capacity in patients with proximal femoral fractures [10, 11]. Exploring the association of these three metabolomic scores with outcomes after COVID-19 infection will provide a broader and more insightful understanding of heterogeneity in disease course.

In this exploratory study, we examined the associations between individual metabolic features and three metabolomic scores with in-hospital disease course, long-term recovery, and overall survival after COVID-19 infection in the adult population. By using well-standardized ^1^H-NMR metabolomics assays and the MetaboHealth, MetaboAge, and Infectious Disease Scores, this study contributes to understanding heterogeneity in key COVID-19 outcomes.

## Methods

### Study population

This study uses data from patients admitted to the Jeroen Bosch Hospital and the Bernhoven Hospital between March and May 2020 during the first wave of COVID-19. The flow chart of inclusion can be seen in Supplementary Fig. 1. All patients hospitalized with a suspected COVID-19 infection were screened for eligibility. Patients were included if they were 18 years or older and if the COVID-19 diagnosis was confirmed with a positive polymerase chain reaction (PCR) test via a nasopharyngeal swab or if a clinical diagnosis was made (based on chest imaging, symptom profile, exposure and exclusion of other causes. This study was not subject to the Medical Research Involving Human Subjects Act (WMO) in the Netherlands, and was reviewed by the institutional review board (IRB) of the Radboud university medical center (file numbers 2020–2923 and 2020–6344). According to the IRB only oral consent was required. After information was provided by the local investigator, oral informed consent was obtained from all of the patients or their relatives included in the cohort and documented in their electronic medical records. Metabolomic profiling was performed on the blood samples of 357 patients. After excluding 11 samples from analysis due to low sample quality and failed profiling, the final study population consisted of data from 346 patients. For the survival analysis, five additional cases were omitted due to missing or unreliable survival time data. Patients older than 60 were contacted by phone between 2 and 2,5 years after admission and asked to give informed oral consent to participate in a follow-up questionnaire about their long-term recovery. Of the 255 eligible patients, 97 gave oral consent and participated in the telephone follow-up.

### Metabolic profiling

Blood samples were collected within 24 h of admission and stored at −80 °C in both hospitals until metabolic profile analysis. The metabolomic profile was measured using a high-throughput ^1^H-NMR platform from Nightingale Health Ltd, platform version 2020. The high throughput nature of ^1^H-NMR metabolomics provides the opportunity to obtain rich metabolic profiles in an affordable and reproducible manner at a biobank scale level. The Nightingale ^1^H-NMR platform is often used as a commercial supplier in research and to measure metabolic profiles in large cohort studies. This panel quantifies 250 metabolic features, including cholesterols, triglycerides, fatty acids, fatty acid ratios, apolipoproteins, amino acids, glycolysis-related metabolites, fluid balance, and inflammation-related markers. Many of the features are correlated which is why many studies focus on about 62 of the more independent features. In addition, 39 of the 250 metabolic features are clinically and analytically validated, allowing direct comparison to the results of any other clinically and analytically validated laboratory method. The ‘Infectious Disease Score’ was based on 37 metabolic features, and after selection comprises 25 metabolic features in an algorithm optimized for predicting the risk for severe pneumonia and COVID-19 in the general population of the UK [6]. The ‘MetaboHealth’ score was based on 63 biomarkers, and after selection comprises 14 metabolic features optimized for the prediction of mortality in multiple population-based cohort studies in the EU [5]. MetaboAge comprises 62 metabolic features optimized for predicting chronological age in multiple population and patient-based Dutch cohorts (BBMRI.Nl) [7]. Furthermore, 10 features were shared between all three metabolic scores. The MetaboHealth, MetaboAge, and Infectious Disease Score were generated such that a higher score indicates poorer health outcomes (increased mortality risk, higher biological age, and higher infectious disease risk, respectively). We calculated all scores, for ‘MetaboAge’ version 2.0, using the R package MiMIR [7, 12, 13]. To obtain the age-independent part of the MetaboAge score (ΔMetaboAge) we regressed out chronological age. Finally, we standardized all the metabolic features and scores to allow direct comparisons.

### Outcome measures

The in-hospital disease course was composed as an ordered categorical variable based on four potential outcomes during hospitalization: (1, ‘mild’) admission and recovery without complications or additional treatments; (2, ‘moderate’) receiving oxygen treatment via an oxygenator or ventilator during hospitalization; (3, ‘severe’) suffering from COVID-19 related complications during hospital stay; (4, ‘fatal’) in-hospital or palliative care mortality. These categories were mutually exclusive, and patients were categorized into the worst outcome category that applied to them. Information on COVID-19-related complications was derived from the medical records. These included pulmonary embolism, acute kidney injury, liver function abnormalities, diarrhea, and delirium [14–18].

Patients who gave oral consent to participate in the telephone questionnaires between 2 and 2,5 years after admission were asked about daily functioning, health problems after COVID-19 and admission, and current mental, social, and physical health. Health problems after COVID-19 include shortness of breath, fatigue, concentration or thinking problems, memory issues, and problems with physical exercise. Patients were listed as having these health problems if they experienced them as new or worsened symptoms after COVID-19 infection. Patients were asked via a standardized questionnaire if they experienced worsening of conditions concerning their physical, social, or mental conditions post-COVID-19 as compared to pre-COVID-19. This questionnaire was standardized across cohorts participating in the COVID-19 Outcomes in Older People (COOP) study and used existing validated questionnaires were possible supplemented with customized items relevant for the aims of this COOP study.

For the survival analysis, mortality was defined as the time in days from admission until the date of death. The date of admission, the date of final contact, and the date of death used for the analysis were derived from the electronic medical records. Patients were censored after they were successfully contacted for the telephone questionnaires. Patients younger than 60, for whom no post-discharge mortality data were collected, were censored at the date of discharge.

### Descriptive variables

Routine care data were extracted from the electronic medical records and entered into an electronic case report form (Castor Electronic Data Capture) based on the ISARIC-WHO COVID-19 CRF [19]. Information collected included demographics, symptoms at admission, laboratory parameters at admission, complications during admission, comorbidities, medication, supportive treatment during admission, and reasons for discharge. Patients were listed as immunocompromised if they suffered from hematological malignancies, auto-immune disease, and/or HIV/AIDS, received stem cell or organ transplantation, or treatment with immunosuppressive medication at admission. The quick Sequential Organ Failure Assessment (qSOFA) score is based on respiratory rate, altered mentation, and systolic blood pressure. It was originally designed to evaluate septic patients, however, it can be used to predict mortality for other diseases [20, 21]. Chronic kidney disease includes chronic deterioration of kidney function whether or not requiring renal replacement therapy. Liver function abnormalities were levels aspartate aminotransferase (ASAT) or alanine aminotransferase (ALAT) five-fold above upper limit of normal levels. Acute kidney injury was defined as a > 25% decrease in eGFR or an eGFR < 60 ml/min. Fever was defined as a temperature higher than 38 °C. Blood pressure medicine includes Angiotensin II receptor blockers and ACE inhibitors. The Clinical Frailty Scale (CFS, range 1–9) was noted only for patients aged 60 or older and categorized as robust (1–3), pre-frail (4–6) and frail (7–9). Patients below the age of 60 were categorized as 1–3 [22, 23]. Obesity was defined as a body mass index (BMI) above 30 kg/m^2^. Restricting policies include non-ICU policy, or non-intubate policy and non-resuscitation policy.

### Statistical analysis

To evaluate whether the two populations from the two hospitals can be assessed as one batch, t-distributed Stochastic Neighbor Embedding (t-SNE) was performed. No differences were observed between the populations [24]. Descriptive statistics were used to compare patients with ‘mild’ and ‘moderate’ in-hospital outcomes with patients with ‘severe’ and ‘fatal’ in-hospital outcomes.

Ordinal regression was conducted to examine the associations between metabolic features and scores with in-hospital disease course. The metabolic features that are clinically and analytically validated or used in the scores were selected for analysis. The proportional hazards assumption of the ordinal regression model was not met. Therefore, additional logistic regression models were used with the outcome in-hospital disease course dichotomized in three ways: ‘mild’ against ‘moderate’, ‘severe’, and ‘fatal’; ‘mild’ and ‘moderate’ against ‘severe’ and ‘fatal’; and ‘mild’, ‘moderate’, and ‘severe’ against ‘fatal’. As age may be an effect modifier, models were stratified by age (< 70 years and ≥ 70 years). The following variables were selected as confounders based on univariable associations with the exposure and outcomes and > 10% change in regression coefficient when added to the model: age, sex, qSOFA at admission, and hospital of admission. To check for multicollinearity, a correlation analysis between all of the confounders was performed and no significant correlations were found. To account for the multiple testing problem, Table 1 (29 tests) and Table 2 in Supplement (62 tests) were controlled for False Discovery Rate. Log-likelihood and Akaike Information Criterion (AIC) were calculated to compare the fit of the models.

**Table 1:** Clinical characteristics of patients admitted to the hospital with COVID-19 infection

	Total (*N* = 346)	Disease outcome:		P -value	Mean Difference [95% CI]	Missing values n(%)
		‘Mild’- ‘moderate’ (*N* = 177)	‘Severe’- ‘fatal’ (*N* = 169)			
Demographics						
Age – mean (SD)	67.9 (12.0)	64.52 (12.1)	71.4 (10.8)	< 0.001*	−6.9 [−9.3; −4.4]	0 (0)
Sex, male – n (%)	234 (67.6)	121 (68.4)	113 (66.9)	0.766	−0.0 [−0.1; 0.1]	0 (0)
Clinical frailty scale – n(%)				< 0.001*	−0.3 [−0.4; −0.1]	4 (1.2)
1–3	244 (70.5)	142 (80.2)	102 (60.4)			
4–5	63 (18.2)	22 (12.4)	41 (24.3)			
6–9	35 (10.1)	11 (6.2)	24 (14.2)			
BMI – mean (sd)	28.9 (5.7)	28.8 (5.7)	29.0 (5.7)	0.831	−0.2 [−1.6; 1.3]	103
Obese– n (%)	90 (26.0)	50 (28.2)	40 (23.7)	0.140	0.1 [−0.03; 0.2]	103
Immunocompromised – n (%)	24 (6.9)	10 (5.6)	14 (8.3)	0.360	−0.0 [−0.1; 0.03]	34
Restricting policies – n (%)	127 (36.7)	46 (26.0)	81 (47.9)	< 0.001*	−0.2 [−0.3; −0.1]	0 (0)
Smoking – n (%)				0.566	0.0 [−0.3; 0.3]	134 (38.7)
No, never	105 (30.3)	55 (31.1)	50 (29.6)			
Yes, former	97 (28.0)	46 (26.0)	51 (30.2)			
Yes, current	10 (2.9)	7 (4.0)	3 (1.8)			
qSOFA score – n (%)				< 0.001*	−0.3 [−0.4; −0.1]	14 (4.0)
0	123 (35.5)	76 (42.9)	47 (27.8)			
1	191 (55.2)	89 (50.3)	102 (60.4)			
2	17 (4.9)	2 (1.1)	15 (8.9)			
3	1 (0.3)	0 (0)	1 (0.6)			
Comorbidities – n (%)						
Autoimmune disease	43 (12.4)	20 (11.3)	23 (13.6)	0.515	−0.0 [−0.1; 0.0]	0 (0)
Diabetes mellitus	84 (24.3)	39 (22.0)	45 (26.6)	0.319	−0.0 [−0.1; 0.0]	0 (0)
Cardiovascular disease	213 (61.6)	103 (58.2)	110 (65.1)	0.187	−0.1 [−0.2; 0.0]	0 (0)
Liver disease	9 (2.6)	3 (1.7)	6 (3.6)	0.278	−0.0 [−0.1; 0.0]	0 (0)
Pulmonary disease	78 (22.5)	42 (23.7)	36 (21.3)	0.589	0.0 [−0.1; 0.1]	0 (0)
Chronic kidney disease	40 (11.6)	14 (7.9)	26 (15.4)	0.030	−0.1 [−0.2; 0.0]	0 (0)
≥ 3 comorbidities	168 (48.6)	77 (43.5)	91 (53.8)	0.054	−0.1 [−0.2; 0.0]	0 (0)
Medication – n (%)						
Immunosuppressive	33 (9.5)	17 (9.6)	16 (9.5)	0.863	0.0 [−0.1; 0.1]	3 (0.9)
Blood pressure lowering	111 (32.1)	55 (31.1)	56 (33.1)	0.918	−0.0 [−0.1; 0.1]	2 (0.6)
Symptoms at admission – n (%)						
Fever	265 (76.6)	141 (79.7)	124 (73.4)	0.198	0.1 [−0.0; 0.1]	1 (0.3)
Cough	255 (73.7)	139 (78.5)	116 (68.6)	0.079	0.1 [−0.0; 0.2]	1 (0.3)
Shortness of breath	237 (68.5)	119 (67.2)	118 (69.8)	0.547	−0.0 [−0.1; 0.1]	1 (0.3)
Fatigue	152 (43.9)	77 (43.5)	75 (44.4)	0.831	−0.0 [−0.1; 0.1]	1 (0.3)
Diarrhea	112 (32.4)	50 (28.2)	62 (36.7)	0.086	−0.1 [−0.2; 0.0]	1 (0.3)
Loss of taste and/or smell	37 (10.7)	27 (15.3)	10 (5.9)	0.012*	0.1 [0.0; 0.2]	1 (0.3)
Confusion	24 (6.9)	8 (4.5)	16 (9.5)	0.068	−0.1 [−0.1; 0.0]	1 (0.3)
Discharge						
Discharge reason – n (%)				< 0.001*	−0.5 [−0.7; −0.3]	10 (2.9)
Clinical improvement	233 (67.3)	153 (86.4)	80 (47.3)			
Patient deceased	68 (19.7)	0 (0)	68 (40.2)			
Transfer to other hospital	32 (9.3)	20 (11.3)	12 (7.1)			
Palliative care	3 (0.9)	0 (0)	3 (1.8)			
Total duration of hospital stay (days)– median (IQR)	4.0 (3.0–9.0)	4.0 (2.0–7.0)	7.0 (4.0–21.0)	< 0.001*	−4.0 [−5.9; −2.1]	13 (3.8)
Complications during admission – n (%)	214 (61.8)	65 (36.7)	149 (88.2)	< 0.001*	0.5 [0.4; 0.6]	0 (0)

Characteristics of the total sample and groups divided by severity of disease course during admission and tested using descriptive statistics: (1, ‘mild’) admission and recovery without complications or additional treatments; (2, ‘moderate’) receiving oxygen treatment via an oxygenator or ventilator; (3, ‘severe’) suffering from COVID-19 related complications during hospital stay; (4, ‘fatal’) in-hospital or palliative care mortality. *BMI* Body Mass Index, *SD* standard deviation, *N* number, *CI* Confidence interval

* Variables that remained statistically significant after controlling for False Discovery Rate

**Table 2:** Association between metabolic features with in-hospital disease course and overall survival after COVID-19 hospitalization

	In-hospital disease course		Overall survival	
	Odds ratio (95% CI)	P-value	Odds ratio (95% CI)	P-value
Cholesterols				
Total cholesterol #^	0.93 (0.75–1.15)	0.501	0.84 (0.68–1.04)	0.113
VLDL cholesterol ^	0.99 (0.80–1.22)	0.913	0.84 (0.67–1.05)	0.129
LDL cholesterol #^	0.87 (0.70–1.08)	0.213	0.78 (0.63–0.96)	0.018
HDL cholesterol ^	1.07 (0.87–1.32)	0.541	1.19 (0.97–1.46)	0.095
IDL cholesterol #^	0.91 (0.74–1.13)	0.387	0.79 (0.64–0.98)	0.033
Fatty acids and triglycerides				
Total triglycerides #^	1.03 (0.84–1.27)	0.770	1.02 (0.81–1.28)	0.857
Total fatty acids	1.08 (0.88–1.34)	0.446	1.08 (0.85–1.36)	0.529
Omega-3 fatty acids #^	0.96 (0.78–1.18)	0.668	0.96 (0.76–1.21)	0.719
Omega-6 fatty acids ^	1.05 (0.85–1.29)	0.679	1.01 (0.80–1.27)	0.933
Polyunsaturated fatty acids #^	1.03 (0.84–1.27)	0.782	1.00 (0.79–1.26)	0.997
Monounsaturated fatty acids #^	1.11 (0.90–1.37)	0.318	1.13 (0.90–1.41)	0.301
Saturated fatty acids ^	1.11 (0.90–1.36)	0.337	1.09 (0.86–1.37)	0.479
Docosahexaenoic acid ^	1.01 (0.83–1.23)	0.940	1.00 (0.77–1.30)	0.986
Linoleic acid ^	1.03 (0.83–1.27)	0.791	0.98 (0.79–1.24)	0.892
Fatty acids ratios				
Ratio omega-3/total fatty acids ^	0.86 (0.70–1.07)	0.170	0.88 (0.70–1.10)	0.259
Ratio omega-6/total fatty acids #^	0.94 (0.76–1.15)	0.524	0.90 (0.73–1.10)	0.307
Ratio polyunsaturated/total fatty acids #@^	0.89 (0.72–1.09)	0.262	0.87 (0.72–1.06)	0.178
Ratio monounsaturated/total fatty acids ^	1.10 (0.90–1.36)	0.359	1.15 (0.94–1.41)	0.171
Ratio saturated/total fatty acids #^	1.07 (0.87–1.32)	0.506	1.03 (0.84–1.26)	0.798
Ratio docosahexaenoic acid/total fatty acids #	0.96 (0.79–1.17)	0.665	0.94 (0.73–1.22)	0.656
Ratio polyunsaturated / monounsaturated fatty acids	0.90 (0.73–1.11)	0.313	0.86 (0.69–1.06)	0.148
Ratio omega-6/omega-3 fatty acids #	1.07 (0.88–1.31)	0.510	1.15 (0.85–1.55)	0.368
Ratio linoleic acid/total fatty acids	0.99 (0.76–1.15)	0.519	0.89 (0.72–1.09)	0.252
Estimated degree of unsaturation ^	0.93 (0.76–1.14)	0.465	0.92 (0.76–1.12)	0.422
Apolipoproteins				
Apolipoprotein B ^	0.95 (0.77–1.17)	0.948	0.80 (0.65–0.99)	0.044
Apolipoprotein A1 ^	1.08 (0.87–1.33)	0.482	1.27 (1.01–1.58)	0.039
Ratio apolipoprotein B/apolipoprotein A #	0.93 (0.75–1.15)	0.481	0.75 (0.60–0.93)	0.010
Amino acids				
Alanine #^	0.95 (0.77–1.16)	0.592	1.10 (0.91–1.34)	0.333
Glutamine ^	0.77 (0.62–0.96)	0.017	1.03 (0.84–1.26)	0.789
Glycine #	0.96 (0.78–1.18)	0.672	0.99 (0.80–1.22)	0.930
Histidine #@^	0.98 (0.79–1.22)	0.847	1.18 (0.97–1.45)	0.106
Isoleucine #@^	0.84 (0.67–1.05)	0.119	0.97 (0.79–1.19)	0.778
Leucine #@^	0.83 (0.67–1.03)	0.097	0.93 (0.74–1.15)	0.496
Valine #@^	0.82 (0.66–1.02)	0.071	0.91 (0.73–1.13)	0.379
Total branched amino acids	0.82 (0.66–1.02)	0.077	0.92 (0.74–1.15)	0.476
Phenylalanine #@^	1.47 (1.17–1.85)	0.001	1.33 (1.14–1.56)	< 0.001*
Tyrosine #^	1.01 (0.81–1.25)	0.966	1.14 (0.94–1.40)	0.192
Glycolysis related metabolites				
Glucose #@^	1.16 (0.94–1.44)	0.169	1.37 (1.16–1.62)	< 0.001*
Lactate #@^	1.26 (1.02–1.56)	0.032	1.38 (1.16–1.63)	< 0.001*
Citrate ^	0.91 (0.74–1.14)	0.420	1.24 (1.00–1.53)	0.045
Fluid balance				
Creatinine #^	1.17 (0.97–1.42)	0.103	1.12 (0.96–1.31)	0.147
Albumin #@^	0.79 (0.62–0.99)	0.039	0.86 (0.70–1.06)	0.158
Inflammation				
Glycoprotein Acetyls #@^	1.21 (0.98–1.49)	0.071	1.22 (0.98–1.51)	0.071
Lipoprotein subclasses				
Total lipids in chylomicrons and extremely large VLDL ^	0.97 (0.79–1.20)	0.794	1.03 (0.83–1.28)	0.772
Total lipids in small HDL^	1.04 (0.84–1.28)	0.730	1.15 (0.89–1.47)	0.282
Total lipids in small LDL ^	0.93 (0.75–1.15)	0.501	0.80 (0.65–0.99)	0.042
Total lipids in small VLDL ^	1.07 (0.86–1.31)	0.554	0.95 (0.76–1.18)	0.627
Total lipids in medium HDL ^	1.12 (0.91–1.37)	0.292	1.36 (1.08–1.70)	0.009
Total lipids in medium LDL ^	0.92 (0.74–1.13)	0.421	0.80 (0.65–0.99)	0.036
Total lipids in medium VLDL ^	0.94 (0.77–1.16)	0.568	0.82 (0.66–1.03)	0.094
Total lipids in very small VLDL ^	1.12 (0.91–1.39)	0.282	0.97 (0.78–1.21)	0.790
Total lipids in large LDL ^	0.92 (0.74–1.13)	0.424	0.80 (0.65–0.99)	0.037
Total lipids in IDl ^	0.96 (0.78–1.18)	0.687	0.82 (0.66–1.02)	0.073
Other lipids				
Phosphatidylcholines and other cholines ^	1.10 (0.89–1.35)	0.389	1.09 (0.86–1.37)	0.497
Phosphoglycerides ^	1.10 (0.89–1.36)	0.364	1.13 (0.89–1.43)	0.315
Total cholines ^	1.08 (0.88–1.33)	0.454	1.08 (0.86–1.36)	0.512
Sphingomyelins ^	1.05 (0.86–1.29)	0.631	0.98 (0.79–1.22)	0.855
Ketone bodies				
Acetoacetate ^	1.10 (0.91–1.34)	0.334	1.12 (0.88–1.43)	0.343
Acetate ^	0.85 (0.68–1.08)	0.183	0.93 (0.76–1.14)	0.488
Lipoprotein particle sizes				
Mean diameter for VLDL particles ^	0.86 (0.70–1.06)	0.153	0.88 (0.70–1.09)	0.235
Mean diameter for LDL particles ^	1.14 (0.93–1.41)	0.218	1.06 (0.86–1.31)	0.608
Mean diameter for HDL particles ^	1.21 (0.97–1.50)	0.089	1.20 (1.01–1.42)	0.039

Odds ratios per 1-SD increase and 95% confidence intervals with in-hospital disease course overall survival. The in-hospital disease course model is adjusted for age, sex, hospital of admission, and qSOFA score at admission. The overall survival model is adjusted for age and sex. *CI* Confidence Interval, *VLDL* very low-density lipoprotein, *LDL* low-density lipoprotein, *HDL* high-density lipoprotein, *IDL* intermediate-density lipoprotein. # = used in Infectious Disease score. @ = used in MetaboHealth. ^ = used in ΔMetaboAge. * = Statistically significant after correction for false discovery rate

Descriptive statistics were used to compare subgroups based on tertiles of the metabolic scores in terms of the long-term recovery outcomes. The subsample with available data on both metabolomics and these outcomes was too small to warrant more advanced statistical analyses. Statistical analyses were done using SPSS version 25.0 and 29.0 (IBM Corp. Released 2017 & 2022. IBM SPSS Statistics for Windows, Version 25.0. & 29.0. Armonk, NY: IBM Corp.).

To examine the association between the selected metabolic features and scores and mortality, Cox proportional hazards regression was used in R (4.3.1) with the survival and survminer packages. Age and sex were included as confounders. Each metabolic score was analyzed both as a continuous variable (z-score standardized) and categorized into tertiles. To assess potential bias introduced by the censoring of younger patients at discharge, we conducted sensitivity analyses excluding patients younger than 60 entirely to assess whether their inclusion affected results.

## Results

### Characteristics of the study population

The final study sample consisted of 346 people, of whom 156 were admitted to the Jeroen Bosch Hospital and 190 to the Bernhoven Hospital. Nearly all patients were had a COVID-19 diagnosis confirmed by PCR testing (n = 342). The mean age of the sample was 67.9 years (SD 12.0), with a predominance of males (67.6%) (Table 1). Most patients were classified as ‘robust’ according to the Clinical Frailty Score (CFS 1–3, 70.5%). Nearly half of the study population had more than 3 comorbidities (48.6%), with the most prevalent comorbidity being cardiovascular disease (61.6%). The most prevalent symptoms at admission were fever, cough, and shortness of breath. The median duration of hospital stay was 4 days (IQR: 3.0–9.0). Two-thirds of patients (67.3%) were discharged home (indicated as ‘clinically improved’), and 71 (20.5%) patients died in-hospital or shortly after in palliative care. Compared with the mild and moderate outcomes groups, the severe and fatal groups had higher mean age, more patients with restricting policies, higher clinical frailty scores and qSOFA scores at admission, more patients with complications during admission, and fewer patients discharged due to clinical improvement (p < 0.001) [25].

### Association between metabolic features and in-hospital disease course and overall survival

None of the 62 metabolic features were statistically significantly associated with in-hospital disease course (Table 2). Phenylalanine, lactate, and albumin showed a trend towards significant associations with in-hospital disease course, however, these became nonsignificant after the false discovery rate method was applied. Increased levels of phenylalanine, glucose, and lactate were the only metabolic features that were significantly associated with overall survival.

### Association between metabolic scores and in-hospital disease course

Higher levels of each of the three metabolic scores were significantly associated with a more severe disease course, with the MetaboHealth score having the strongest association (Table 3). The log-likelihood and AIC of the MetaboHealth and Infectious Disease Score were similar and indicated a better fit to the model than the ΔMetaboAge profile. Additional logistic regression models showed that for each of the three scores, the strongest associations were found when the outcome was dichotomized as mild versus moderate, severe, and fatal (Table 4).

**Table 3:** Associations between metabolic scores and in-hospital disease course

	Odds ratio	Lower 95% CI	Upper 95% CI	P-value	Log Likelihood	AIC
All participants (*n* = 346)						
MetaboHealth	1.61	1.29	2.02	< 0.001	−380.1	776.1
Infectious Disease Score	1.55	1.25	1.93	< 0.001	−381.1	778.3
ΔMetaboAge	1.42	1.16	1.74	< 0.001	−399.1	812.2
Participants aged < 70 years (*n* = 168)						
MetaboHealth	1.89	1.33	2.68	< 0.001	−164.8	345.6
Infectious Disease Score	1.60	1.15	2.23	0.005	−167.4	350.9
ΔMetaboAge	1.73	1.18	2.54	0.005	−168.9	351.8
Participants aged ≥ 70 years (*n* = 178)						
MetaboHealth	1.61	1.18	2.20	0.003	−207.1	430.2
Infectious Disease Score	1.64	1.21	2.23	0.001	−206.4	428.9
ΔMetaboAge	1.26	0.99	1.61	0.058	−212.3	438.6

Odds ratios per 1 standard deviation increase in score and 95% confidence intervals derived from ordinal regression (total *N* = 346). Models are adjusted for age, sex, hospital of admission, and qSOFA score at admission. *CI* Confidence Interval, *AIC* Akaike’s Information Criterion

**Table 4:** Logistic regression models testing the associations between metabolic scores and categories of severe disease outcomes during COVID-19 hospitalization

	Odds ratio	Lower 95% CI	Upper 95% CI	P-value	Log Likelihood	AIC	AUC
Predicting oxygen treatment, COVID-19-related complications and mortality							
MetaboHealth	2.69	1.73	4.17	< 0.001	−76.6	165.1	0.784
Infectious Disease Score	2.29	1.52	3.44	< 0.001	−79.4	170.8	0.782
ΔMetaboAge	1.16	0.75	1.78	0.503	−88.7	187.4	0.526
Predicting COVID-19-related complications and mortality							
MetaboHealth	1.32	1.03	1.69	0.026	−205.7	423.4	0.600
Infectious Disease Score	1.39	1.08	1.79	0.010	−204.8	421.6	0.605
ΔMetaboAge	1.53	1.19	1.96	< 0.001	−213.8	437.7	0.579
Predicting mortality							
MetaboHealth	1.86	1.33	2.61	< 0.001	−135.7	283.4	0.663
Infectious Disease Score	1.63	1.18	2.26	0.003	−138.4	288.8	0.639
ΔMetaboAge	1.38	1.06	1.78	0.015	−140.0	292.1	0.559

Odds ratios per 1-SD increase in metabolic scores and 95% confidence intervals with in-hospital disease course are estimated using ordinal regression. Models are adjusted for age, sex, hospital of admission, and qSOFA score at admission. *CI* Confidence Interval, *AIC* Akaike’s Information Criterion, *AUC* Area Under the Curve from Receiver Operator Characteristics (ROC) curve. Groups for severity of outcome during COVID-19 hospitalization: (1, ‘mild’) admission and recovery without complications or additional treatments; (2, ‘moderate’) receiving oxygen treatment via an oxygenator or ventilator; (3, ‘severe’) suffering from COVID-19 related complications during hospital stay; (4, ‘fatal’) in-hospital or palliative care mortality. * = *p* < 0.05

To test if the association with outcomes is stronger among relatively older individuals with often pre-existing and sometimes undetected morbidities, we performed age-stratified analyses. 168 (48.6%) patients were younger than 70 years and 178 (51.4%) were 70 years or older. Age-stratified analyses showed that the associations with in-hospital disease course were somewhat stronger for MetaboHealth and ΔMetaboAge in the younger age group than in the older age group, although confidence intervals were overlapping (Table 3). The associations between the Infectious Disease Score and disease course were similar in the two age groups.

### Association between metabolic scores and long-term recovery

**Fig. 1:**
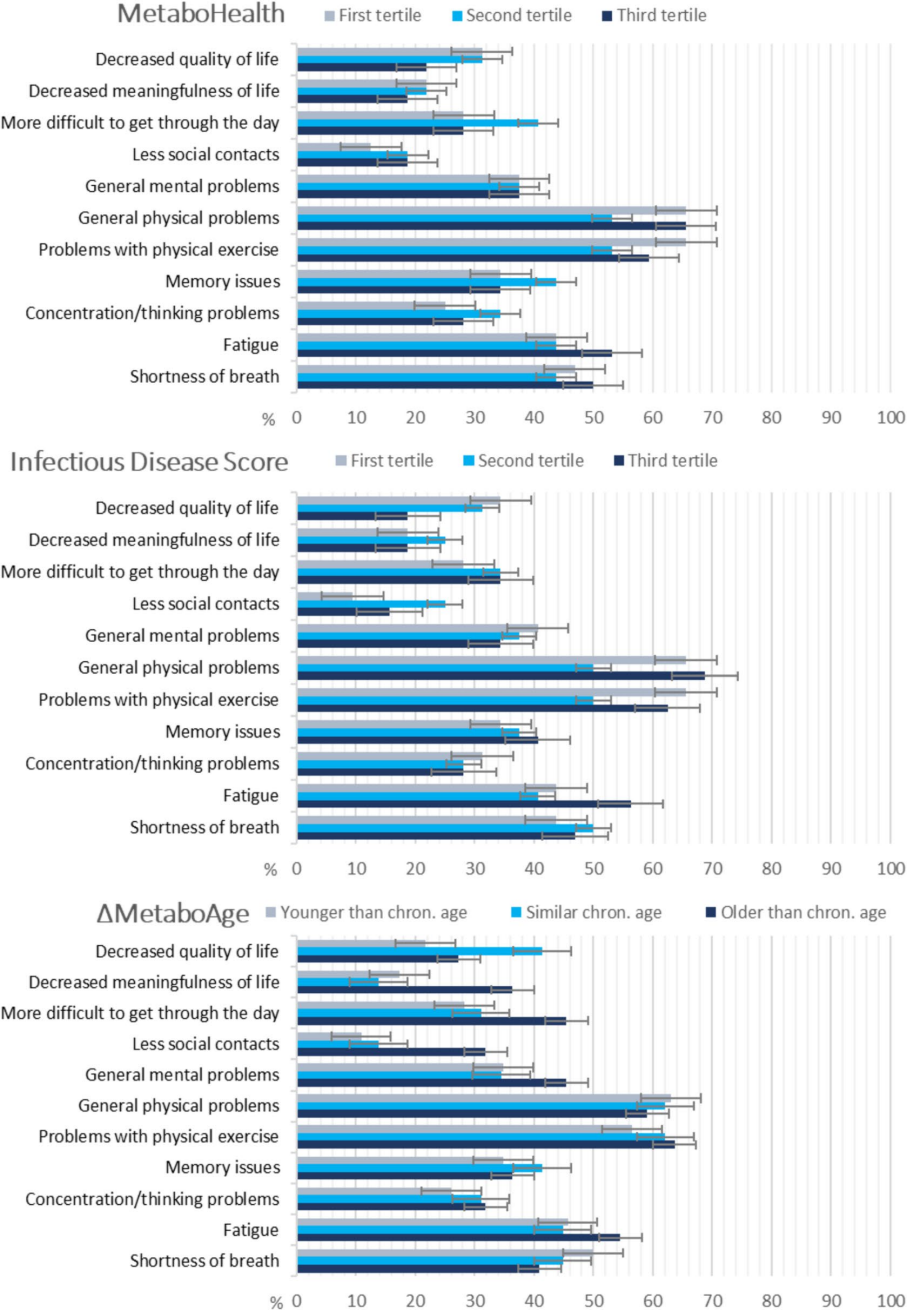
Prevalence of experiencing new or worsening health problems post-COVID-19. Error bars reflect standard error. The MetaboHealth score and Infectious Disease Score were split into equal tertiles. The ΔMetaboAge score was split into patients with younger (< 5 years), similar ([−5,5]), and older (> 5 years) metabolic age compared to chronological age

The health problems that patients most frequently reported post-COVID-19 were problems with physical exercise, fatigue, and shortness of breath (Fig. 1). No consistent trends in the prevalence of health problems were visible across tertiles of the metabolic scores. Patients most often experienced a decline in physical condition, followed by a decline in mental condition and trouble with getting through the day (Fig. 1). Again, no consistent trends were visible between the metabolic scores in the prevalence of decline in conditions.

### Association of metabolic scores with overall survival

**Fig. 2:**
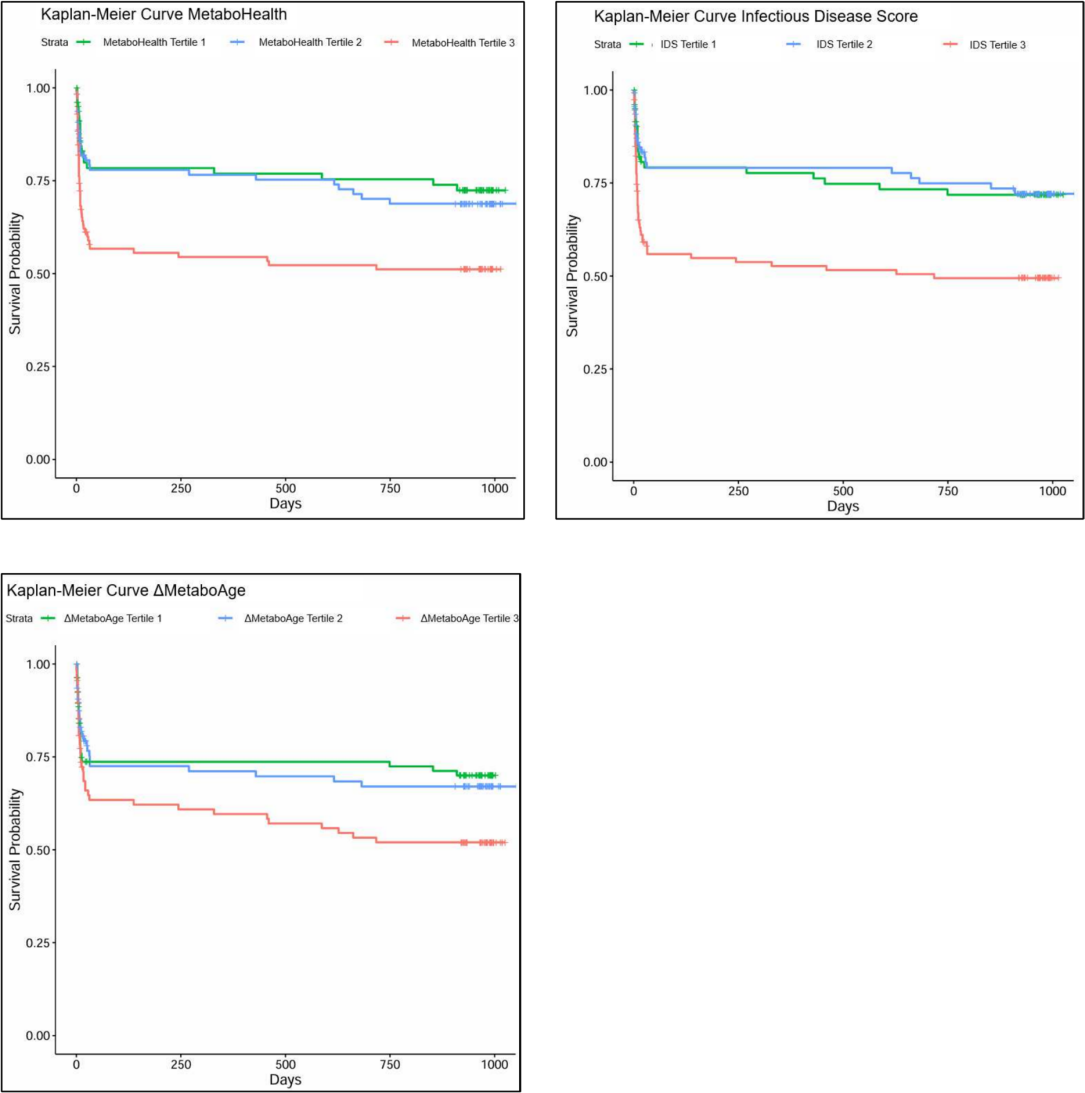
Association of metabolic scores in tertiles with overall survival. Kaplan–Meier curves of cumulative survival of patients using cox proportional hazards regression (*n* = 327). The three scores were categorized into tertiles (low, moderate, and high). Models are adjusted for age and sex

All three metabolic scores were significantly associated with overall survival after COVID-19 infection (Fig. 2; Supplementary Table 1). Each 1-standard deviation (SD) increase in MetaboHealth, Infectious Disease Score, and ΔMetaboAge was associated with a 55% (HR = 1.55, 95% CI: 1.27–1.90, p < 0.001), 53% (HR = 1.53, 95% CI: 1.24–1.89, p < 0.001), and 33% (HR = 1.33, 95% CI: 1.14–1.56, p < 0.001) higher mortality risk, respectively. Patients in the highest tertile of each score had a substantially increased risk of mortality compared to those in the lowest tertile (HR = 2.22 for MetaboHealth, HR = 2.30 for Infectious Disease Score, and HR = 1.87 for ΔMetaboAge, all p ≤ 0.009). Sensitivity Analyses, excluding participants younger than 60, confirmed the robustness of these associations, with hazard ratios remaining within a similar range (Supplementary Table 2).

## Discussion

After performing metabolomic profiling of 346 patients admitted to two hospitals in the Netherlands with a COVID-19 infection, we examined associations of both individual metabolites and the sum scores MetaboHealth, Infectious Disease Score and MetaboAge, with in-hospital disease course, long-term recovery, and overall survival in patients admitted for COVID-19 infection. None of the 62 individual metabolites were significantly associated with the in-hospital disease course, whereas all three metabolic scores showed similar levels of significant associations. The metabolites phenylalanine, lactate, and glucose, as well as all three metabolic scores were associated with overall survival. Those in the highest tertiles of MetaboHealth, Infectious Disease Score and MetaboAge had the highest mortality risks. No patterns could be observed across the three metabolic scores in their associations with outcomes of long-term recovery.

This study highlights the value of metabolic scores over single metabolites, since all three metabolic scores, each designed for different outcomes, convert mostly non-significant single metabolic features into an algorithm significantly indicative of severe outcomes during COVID-19 hospitalization and mortality after infection. Metabolomic profiling provides the opportunity to search for patterns of metabolites leading to a better understanding of metabolic and physiological functioning. From the quality control of Nightingale, it was observed that many of the individual metabolic features were shifted compared to a normal population. These include decreased cholesterol and lipid levels, a skewed fatty acid balance, and increased albumin and glycoprotein acetyls levels. The shift compared to the normal population can most likely be attributed to the average older age and acute infection status of our study sample. This shift might explain why we find no associations between single metabolic features and severe in-hospital outcomes. We also see little overlap between metabolites associated with mortality in the population at large in the original MetaboHealth paper and the admitted COVID-19 patients here, except for glucose, alanine, and phenylalanine [5]. The association between glucose and all-cause mortality may be attributed to a loss in glycemic control. Our findings are in line with other studies that have shown that elevated glucose at admission in the context of COVID-19 is associated with increased mortality [26–29]. Previous studies have shown phenylalanine to be a disease severity marker related to respiratory distress, independent of the magnitude of the inflammatory state. Several pathways have been suggested to explain the increase of phenylalanine during severe COVID-19 infection, including release due to the promotion of muscle breakdown by inflammatory cytokines and the need for phenylalanine in the production of viral proteins and assembly of viral particles [30, 31]. Lactate is the end product of anaerobic glycolysis, which has been suggested to be upregulated during severe infection by cytokine-induced tissue damage and the subsequent release of lactate dehydrogenase, the enzyme responsible for the formation of lactate, which has been associated with increased mortality in the context of COVID-19 [32, 33]. These collective findings may suggest that glycemic control and respiratory distress play a role in explaining the heterogeneity in disease course, specifically mortality risk. However, all three metabolic scores were also associated with these outcomes. These three scores, and especially MetaboHealth, indicate overall vulnerability (e.g. frailty and cognitive decline) and are associated with a range of inflammatory proteins (D. Bizzarri et al., personal communication, June 2024) [9, 34]. It may also be that overall biological frailty/vulnerability drives disease heterogeneity rather than just the glycemic and respiratory systems. This explanation is supported by studies that show associations between other measures of frailty and mortality in COVID-19 patients [35]. Based on the current findings, no definite conclusions can be drawn, but these findings help direct future research in this area.

Previously, the MetaboHealth profile was found to be associated with biological age, frailty, and cognitive and functional decline better than MetaboAge [8, 9]. The Infectious Disease Score has been associated with severe COVID-19 and pneumonia when measured a decade before infection and when measured during a COVID-19 infection [36, 37]. In these latter study a comparison to MetaboHealth was not performed. Based on these previous findings, we had expected that MetaboHealth would be more indicative of frailty and Infections Disease Score would be more indicative of infection severity. However, the predictive value (expressed as ORs and AUCs) of the two scores were in the same range for all in-hospital outcomes and overall survival (Tables 3 and 4 and Supplementary Table 1), suggesting that either the two scores measure the same thing or that both frailty and infection severity play have similar impacts on the outcomes. Given the overlap in metabolites included in the two scores, it seems likely that both scores capture similar or related aspects of physical frailty. It may be that the Infectious Disease Score reflects molecular signals of low-grade inflammation that increases the severity of both infectious and chronic diseases. The MetaboAge score indicates the risk of future cardiovascular disease, mortality, and functionality in the oldest old [7]. Here, we show that all three scores are associated with severe in-hospital disease course and overall survival after a COVID-19 infection. Since these scores are based partly on the same biomarkers, the similarities in results between the scores are expected. The prediction of outcomes following infection is potentially harsher among older individuals with often existing (undetected) morbidity. Therefore, we performed additional stratified analysis for patients younger and older than 70. Associations between MetaboHealth and Infectious Disease Score and in-hospital mortality were in the same range for both age-groups. However, for MetaboAge, a stronger association was found in the younger age group than in the older age group, in which the association was no longer statistically significant (Table 2). Since especially MetaboHealth, and to a lesser extent ΔMetaboAge, have been previously associated with frailty [38], this might indicate that frailty plays a larger role in younger patients in the context of COVID-19 and less so in the older age group. However, other studies found associations in older adults between frailty and mortality risk in COVID-19 patients making this explanation less likely [39]. An alternative explanation is that individual metabolites are less affected by the acute infection in older patients than in younger patients as a result of less responsive immune and metabolic systems. The metabolites present in MetaboAge that are absent in MetaboHealth include glutamine, citrate, creatinine and tyrosine, for example, and also the relevant low grade inflammation marker GlcA, so prominent in MetaboHealth. Perhaps at younger age these are metabolites that diverge more by infection and inflammatory responses than in older individuals. GLC A marks mortality (hence prominent in MetaboHealth) whereas it does not mark chronological age. According to the theory of complex dynamic systems, a healthy, resilient system adequately responds to stressors, resulting in greater variation in signals of that system [40]. Applied to the current context, this would mean that greater variation in metabolites reflects a better response of the immune and metabolic systems to the stressor, being COVID-19. Vice versa, lower variation in metabolic scores in older patients may reflect a compromised immune or metabolic response. This is in line with the literature suggesting that immunosenescence suppresses an effective immune response in older adults [41]. However, as metabolites were measured at one time-point only, within-person variation over time could not be examined in this study. Given the inconsistent findings within our study and across studies, no strong conclusions can be drawn regarding the role of age in the association between metabolomic scores and COVID-19 outcomes.

No clear patterns could be observed across the three metabolic scores in their associations with recovery outcomes. These data were available only for a subsample of the older patients (60 + , n = 97). Since higher metabolic scores reflect less healthy states, one would expect to observe higher proportions of self-reported health decline or health problems in the highest tertiles of metabolic scores. This was true for some but not all outcomes. For example, the percentage of patients reporting problems with physical exercise was higher in the lowest than in the highest tertile of MetaboHealth, and similar percentages reported general physical and mental health problems. Note that patients were asked to report self-perceived changes in health 2–2,5 years after COVID-19 relative to before COVID-19. This absence of a clear pattern in findings may be explained by these older patients in the highest tertile already experiencing more health problems prior to COVID-19 and thus perceiving less worsening over time. In addition, other factors than COVID-19 may play a more important role, such as general aging-related decline or other illnesses. Given the relatively small sample size and mixed findings, no firm conclusions can be drawn and replication in larger samples is recommended.

A strength of this study is the clinically relevant and representative sample. This is supported by similar sample characteristics in the current sample compared to those in two other cohorts of hospitalized COVID-19 patients [42, 43]. A limitation of this study was the relatively small sample size, particularly for the long-term recovery outcomes. All ages were included to improve statistical power, however, for younger adults, the CFS was not noted in their medical records, and data on long-term outcomes were not collected. After sensitivity analysis, we partly overcame this issue by imputing missing values based on the assumption that the younger adults were not frail and survived the period of our survival analysis. Although this is likely true for most patients, it will have led to some misclassification and potentially underestimation of true associations. Our study only included individuals with COVID-19 admitted to the hospital, which means the results represent hospitalized COVID-19 patients only. Including mild or asymptomatic cases would have resulted in a broader range of COVID-19 severity and potentially stronger associations and broader generalizability of the findings. Our definition of disease course was based on presence of oxygen support, complications or in-hospital death. Invasive oxygen support may be seen as ‘more severe’ than non-invasive oxygen support. However, adding another category would result in few cases per category and limit the statistical power. Moreover, 80% of cases with invasive ventilation also had complications or died during admission. Therefore, these cases were already assigned to the severe or fatal disease course categories and adding another category is unlikely to change the current findings. Another limitation was that metabolites were measured at one timepoint only and during the acute phase of the infection. We could therefore not differentiate whether shifts in metabolites were driven by underlying pre-existing metabolic dysregulation or by the bodies response to the COVID-19 infection. As described in the methods and results, the proportional odds assumption of the ordinal regression model was not met, meaning that the odds ratio was not constant across the categories of the outcome variable. We accounted for this violation by conducting additional logistic regression models to calculate the associations between the categories of severe outcomes during COVID-19 hospitalization. The results were in the same direction as for the ordinal regression models, with the addition that the strongest association of metabolomic scores with disease course was found when dichotomized as mild versus moderate, severe, and fatal (Table 4). Lastly, the aim of the current study was to examine whether or not there is an association between metabolomic values and sum scores and the disease course in patients with COVID-19 infections. If these findings can be verified in another sample, an interesting direction for future research is to examine the added predictive value of these metabolomic scores beyond routinely available clinical information.

In conclusion, this study highlights the value of metabolic profiling and using metabolic scores due to the strong associations of the metabolic scores as opposed to the metabolic features. We show that all three metabolic scores are associated with in-hospital disease course and survival, but not long-term recovery. Our findings suggest that either glycemic control and respiratory distress and/or overall biological frailty or vulnerability may explain the heterogeneity in disease course and mortality risk, however, further research is required to confirm this.

## Supplementary Information

Below is the link to the electronic supplementary material.

Supplementary file1 (DOCX 23 KB)

Supplementary file2 (PDF 97 KB)

## Data Availability

We are in the process of storing our data in a repository (https://data.ru.nl/collections/ru/rumc/clinico_t0000030a_dsc_352, ‘Clinico/ Biomarco database’). Upon request, reviewers can be given access to the digital research environment where our data is currently stored if they wish to check our files and codes. For more information, please contact Jacobien Hoogerwerf (Jacobien.Hoogerwerf@radboudumc.nl).
