# Supplementary material for: Associations between metabolomic scores and clinical outcomes in hospitalized COVID-19 patients
Source: GeroScience. 2025 Mar 11;47(3):4395–411. doi: 10.1007/s11357-025-01591-z (PMC12181578; doi:10.1007/s11357-025-01591-z)
Supplement: Supplementary file 1 — Supplementary file1 (DOCX 23 KB) [file 11357_2025_1591_MOESM1_ESM.docx]

**Associations between metabolomic scores and clinical outcomes in hospitalized COVID-19 patients**

Jens A. Venema, BSc,^1^ Anna Kuranova, PhD,^1^ Daniele Bizzarri, MSc**,**^2^ Simon P. Mooijaart, MD PhD,^3,4^ Angele P.M. Kerckhoffs, MD PhD, ^5^ Kitty Slieker, MD,^6^ Evertine J. Abbink, PhD,^7^ Harmke A. Polinder-Bos, MD PhD,^8^ Eline Slagboom, PhD ^2^ Geeske Peeters, PhD,^1,9*^ on behalf of the COOP consortium

^1^ Department of Geriatric Medicine, Radboud university medical center, Nijmegen, the Netherlands

^2^ Department of Biomedical Data Sciences, Section of Molecular Epidemiology, Leiden University Medical Center, Leiden, the Netherlands

^3^ Department of Internal Medicine, Section of Gerontology and Geriatrics, Leiden University Medical Center, Leiden, the Netherlands

^4^ LUMC Center for Medicine for Older People, Leiden University Medical Center, Leiden, the Netherlands

^5^ Department of Internal medicine, Department of Geriatrics, Jeroen Bosch Hospital, Den Bosch, the Netherlands

^6^ Department of Internal Medicine, Bernhoven Hospital, Uden, the Netherlands

^7^ Department of Internal Medicine, Radboud university medical center, Nijmegen, the Netherlands

^8^ Section of Geriatrics, Department of Internal Medicine, Erasmus MC University Medical Centre, Rotterdam, the Netherlands

^9^ Radboudumc Alzheimer Centre, Radboud university medical center, Nijmegen, the Netherlands

Address for correspondence: Dr Geeske Peeters, Radboud university medical centre, Geert Grooteplein Zuid 10, Route 925, Postbus 9101, 6500 HB Nijmegen, The Netherlands, T:+31(0)243616772, E: [geeske.peeters@radboudumc.nl](mailto:geeske.peeters@radboudumc.nl)

**Collaborators of the COOP consortium**

Simon P. Mooijaart^1,2^, Jacobijn Gussekloo^1,2,3^, Harmke A. Polinder-Bos^4^, Karel G.M Moons^5^, Maarten van Smeden^5^, Geeske Peeters^6^, René J.F. Melis^6^, Petra J.M. Elders^7^, Jan Festen^8^, Geert-Jan Geersing^9^, Hannah M. la Roi-Teeuw^9^, Carline J. van den Dries^9^

1 Department of Internal Medicine, Section of Geriatrics and Gerontology, Leiden University Medical Center, Leiden, The Netherlands

2 LUMC Center for Medicine for Older People, Leiden University Medical Center, Leiden, The Netherlands

3 Department of Public Health and Primary Care, Leiden University Medical Center, Leiden, The Netherlands

4 Division of Geriatric Medicine, Department of Internal Medicine, Erasmus MC, University Medical Center Rotterdam, Rotterdam, The Netherlands

5 Julius Center for Health Sciences and Primary Care, University Medical Center Utrecht, Utrecht University, Utrecht, the Netherlands

6 Department of Geriatric Medicine, Radboud University Medical Center, Nijmegen, The Netherlands

7 Department of General Practice, Amsterdam Public Health Research Institute, Amsterdam University Medical Centre, location VU, Amsterdam, The Netherlands

8 KBO-PCOB, Etten-Leur, The Netherlands.

9 Department of General Practice and Nursing Science, Julius Center for Health Sciences and Primary Care, University Medical Center Utrecht, Utrecht University, The Netherlands

**Supplemental material - Associations between metabolomic scores and clinical outcomes in hospitalized COVID-19 patients**

*Intended for publication as an online data supplement*

**Supplementary Table 1.** Association of metabolic scores with overall survival.

| Predictor | **Hazard ratio** | **Low 95% CI** | **High 95% CI** | **P-value** |
| --- | --- | --- | --- | --- |
| **MetaboHealth (n = 339)** | | | | |
| Continuous (Z-score) | 1.55 | 1.27 | 1.90 | <0.001 |
| Lowest tertile | Reference category | | | |
| Moderate tertile | 1.20 | 0.69 | 2.11 | 0.52 |
| Highest tertile | 2.22 | 1.34 | 3.69 | 0.002 |
| **Infectious Disease Score (n = 338)** | | | | |
| Continuous (Z-score) | 1.53 | 1.24 | 1.89 | <0.001 |
| Lowest tertile | Reference category | | | |
| Moderate tertile | 1.23 | 0.68 | 2.22 | 0.49 |
| Highest tertile | 2.30 | 1.39 | 3.82 | <0.001 |
| **ΔMetaboAge (n = 341)** | | | | |
| Continuous (Z-score) | 1.33 | 1.14 | 1.56 | <0.001 |
| Lowest tertile | Reference category | | | |
| Moderate tertile | 1.27 | 0.76 | 2.13 | 0.35 |
| Highest tertile | 1.87 | 1.16 | 3.02 | 0.009 |

*Hazard ratios (HR) and 95% confidence intervals (CI) for overall survival, estimated using Cox proportional hazards regression. Continuous values represent hazard ratios per 1-standard deviation (SD) increase in metabolic scores, while categorical values compare moderate and high tertiles to the lowest tertile (reference category). All models are adjusted for age and sex. Significance threshold: p < 0.05.*

**Supplementary Table 2.** Sensitivity analyses: Association of metabolic scores with overall survival excluding participants under 60 years of age.

| Predictor | **Hazard ratio** | **Low 95% CI** | **High 95% CI** | **P-value** |
| --- | --- | --- | --- | --- |
| **MetaboHealth** (n=252) | | | | |
| Continuous (Z-score) | 1.53 | 1.25 | 1.88 | <0.001 |
| Lowest tertile | Reference category | | | |
| Moderate tertile | 1.28 | 0.73 | 2.26 | 0.39 |
| Highest tertile | 2.21 | 1.31 | 3.72 | 0.002 |
| **Infectious Disease Score** (n=251) | | | | |
| Continuous (Z-score) | 1.56 | 1.27 | 1.93 | <0.001 |
| Lowest tertile | Reference category | | | |
| Moderate tertile | 1.42 | 0.77 | 2.26 | 0.26 |
| Highest tertile | 2.57 | 1.52 | 4.37 | <0.001 |
| **ΔMetaboAge** (n=254) | | | | |
| Continuous (Z-score) | 1.33 | 1.14 | 1.56 | <0.001 |
| Lowest tertile | Reference category | | | |
| Moderate tertile | 1.29 | 0.76 | 2.19 | 0.34 |
| Highest tertile | 1.92 | 1.17 | 3.14 | 0.009 |

*Hazard ratios (HR) with 95% confidence intervals (CI) for overall survival, estimated using Cox proportional hazards regression. Continuous values represent the hazard ratio per 1-standard deviation (SD) increase in metabolic scores, while categorical values compare the moderate and high tertiles to the lowest tertile (reference category). All models are adjusted for age and sex. Statistical significance is indicated by p < 0.05.*
