# Supplementary material for: Associations between metabolomic scores and clinical outcomes in hospitalized COVID-19 patients
Source: GeroScience. 2025 Mar 11;47(3):4395–411. doi: 10.1007/s11357-025-01591-z (PMC12181578; doi:10.1007/s11357-025-01591-z)
Supplement: Supplementary file 2 — Supplementary file2 (PDF 97 KB) [file 11357_2025_1591_MOESM2_ESM.pdf]

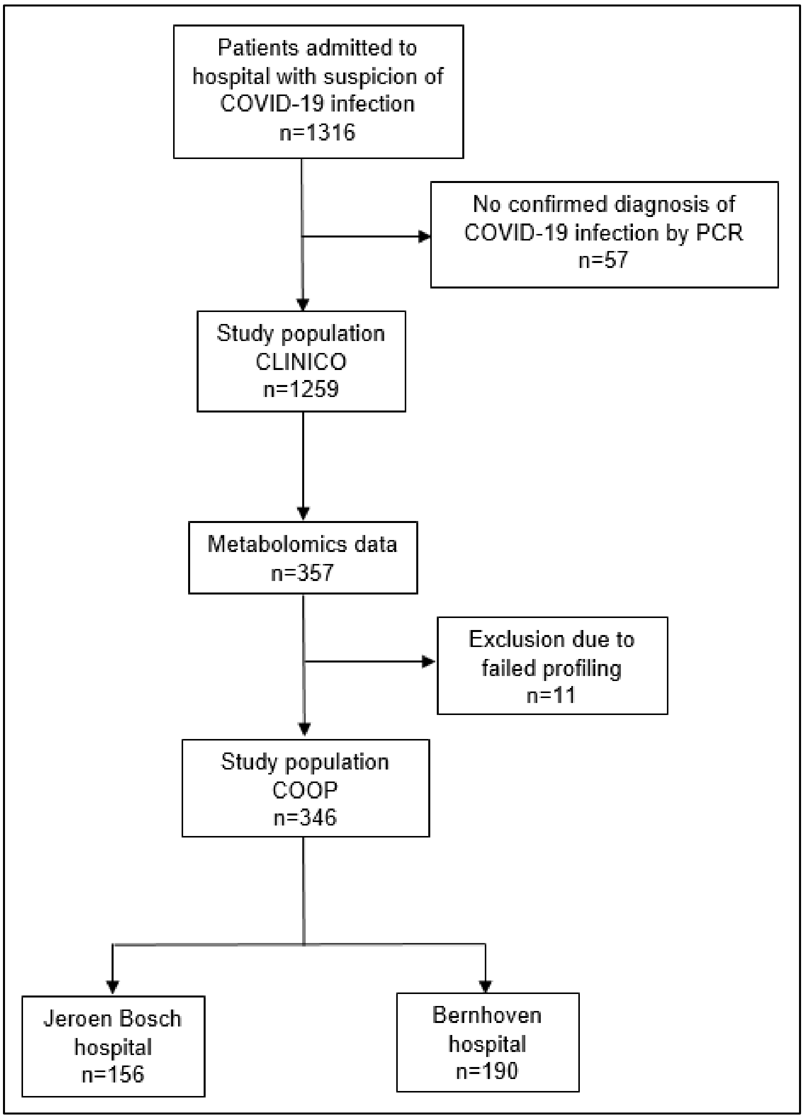

Supplementary Figure 1. Flow chart of inclusion of study participants.
Overview of patients from the Jeroen Bosch Hospital and Bernhoven Hospital included for metabolic profiling.
